# From a research trial to routine practice: stakeholders’ perceptions and experiences of referrals to the National Exercise Referral Scheme (NERS) in Wales

**DOI:** 10.1186/s12913-021-07266-7

**Published:** 2021-11-13

**Authors:** Kelly Morgan, Jennifer Lewis, Jemma Hawkins, Graham Moore

**Affiliations:** 1 Centre for Development, Evaluation, Complexity and Implementation in Public Health Improvement (DECIPHer), School of Social Sciences, Cardiff University, 1-3 Museum Place, CF10 3BD Cardiff, UK; 2 School of Medicine, Cardiff University, Neuadd Meirionnydd, CF14 4YS Cardiff, UK

**Keywords:** Physical activity, Exercise Referral, Referral system, Primary care, UK

## Abstract

**Background:**

Over ten years on from a randomised controlled trial and subsequent national roll-out, the National Exercise Referral Scheme (NERS) continues to be routinely delivered in primary care across Wales, UK. Few studies have revisited effective interventions years into their delivery in routine practice to understand how implementation, and perceived effects, have been maintained over time. This study explores perceptions and experiences of referral to NERS among referrers, scheme deliverers and patients.

**Methods:**

Individual, semi-structured interviews were conducted with 50 stakeholders: scheme referrers (*n* = 9); scheme deliverers (*n* = 22); and referred patients (*n* = 19). Convenience sampling techniques were used to recruit scheme referrers and purposive sampling to recruit scheme deliverers and patients. Thematic analysis was employed.

**Results:**

Analyses resulted in five key themes; referrer characteristics, geographical disparities in referral and scheme access, reinforcements for awareness of the scheme, patient characteristics and processes and context underpinning a referral. Overall there was a high concordance of views between all three stakeholder groups and barriers and facilitators were found to be entwined within and across themes. Referral barriers persisting since the earlier trial included a lack of consultation time and a lack of referral feedback. Newly identified barriers included a lack of scheme awareness and a referral system perceived to be time intensive and disjointed. Key referral facilitators included patient self-referrals, a growing scheme reputation and promotional activities of scheme deliverers.

**Conclusions:**

Findings provide evidence that could inform the further development of NERS and wider exercise referral schemes to ensure the referral process is timely, efficient and equitable.

**Supplementary Information:**

The online version contains supplementary material available at 10.1186/s12913-021-07266-7.

## Introduction

Physical activity is a modifiable risk factor for the prevention of adverse health outcomes [1]. That said, nearly a quarter of adults are inactive worldwide [2], and physical inactivity is currently a leading risk for non-communicable diseases. In the United Kingdom (UK), one third of men and almost half of women are insufficiently active for good health [3].

Over the past two decades, primary care settings have adopted Exercise Referral Schemes (ERS) as a strategy for managing physically inactive individuals [4, 5]. ERS involve the referral of a patient from a health professional to a structured and tailored exercise programme and usually last 10-12 weeks [6, 7]. Improvements in long-term physical activity levels [8] and health outcomes [8–11] however, have typically been shown among ERS lasting 16 or more weeks. In Wales, UK, a national ERS continues to be delivered across all local authorities (LAs), following positive impacts found in an earlier randomized controlled trial (RCT) [8]. More than ten years later, this evidence-based ERS, formally known as NERS (National Exercise Referral Scheme), has received over 80,000 referrals [12]. NERS comprises a supervised 16-week exercise programme for patients over aged 16 years who have, or are at risk of developing, a chronic disease. Patients are typically referred into the scheme by a general practitioner (GP) or alternative health professional as a way to support longer-term lifestyle changes to improve patient health and wellbeing.

The referral process signifies the starting point of a patient’s journey into an ERS and as such, can have a profound impact on both the referral and uptake rate to any one scheme. Research among patient and referrer groups has shown contrasting perspectives, with an eagerness for referral among patients yet a reluctance among health practitioners to refer sedentary individuals [13]. Wider studies have demonstrated that referral decisions are entangled with contextual barriers such as a lack of consultation time and competing patient needs, which can lead to unsystematic referrals and a lack of priority for practitioners to partake in patient physical activity behavior change [14]. Earlier research as part of the process evaluation embedded within the RCT of NERS highlighted that health professionals acknowledged the importance of physical activity in conjunction with a high volume of patients requesting a referral [15], yet reluctance among some health professionals to promote physical activity via primary care was found [16]. Specifically, referrer physical activity levels and subjective judgements on patient motivation levels were noted as influential factors for referral decisions while geographic isolation, ambiguous patient selection criteria and a lack of scheme feedback were cited as key referral barriers [16].

Our recent findings found a widening of inequality in patient referral and uptake of NERS since the earlier RCT [12]. Over a ten-year period, we observed a downward trend for the referral of patients in the most deprived groupings, coupled with a marked pattern of disparity in uptake rates. Patients referred for mental health reasons were also found to be least likely to take up the scheme compared to patients referred for Coronary Heart Disease reasons, echoing earlier reports of uptake among ERS and wider physical activity provision. Identifying potential reasons for these observations and revisiting the previously identified referral barriers remains a crucial step to ensuring future access to and use of NERS is equitable. Furthermore, among the small number of aforementioned studies attempting to identify the factors impacting upon referral decisions [13–16], data are limited to the viewpoint of one stakeholder group, with studies typically focusing on the perspectives of referring health professionals. One study gathering perspectives from referring health professionals and exercise professionals noted tensions with partnership working, as some referrers were considered advocates and others perceived as disinterested in the scheme [17]. In order to consider broader contextual factors, it is important to gather the perspectives of wider stakeholder groups including that of the service user.

More than ten years since the earlier RCT and process evaluation, NERS continues to be sustained in routine practice. The present study reports findings from qualitative interviews with scheme referrers, patients and scheme deliverers. Interviews were used to explore perceptions and experiences of referral to NERS with a view to uncover referral barriers and facilitators which have emerged or persisted over time.

## Methods

### Setting

NERS is delivered across all 22 LAs in Wales and has received a total of 83,598 generic pathway patient referrals between 2007 and 2017 [12]. In order to attend the programme, a patient requires a referral from a health professional to a community-based facility within their LA whereby they undertake an initial scheme consultation. Typically, the referring health professional for generic pathway patients is a GP but in more recent years wider health professionals (e.g. physiotherapists) have started to refer to the scheme, reflecting the existing practice of wider UK-based ERS [18]. This project sits within a wider study of the long term implementation of NERS in Wales [12].

### Recruitment strategy

In each LA a coordinator oversees delivery of the scheme. The coordinator of each LA in Wales was invited to take part in the study and are referred to herein as ‘scheme deliverers’.

Scheme participants were recruited from four purposively sampled LAs, chosen to represent trends in referral numbers into NERS over the past five years (i.e. highest referral rate area, lowest referral rate area, area with largest increase in referrals and area with smallest increase in referrals). Coordinators and exercise professionals helped to facilitate the recruitment of scheme participants within their LA. Specifically, a researcher was invited to attend either group- or gym-based sessions within each LA. During four group sessions, the researcher addressed the whole group (on average 20 participants per session) to introduce the study and opportunity to participate. Within two gym-based sessions, the researcher approached scheme participants individually (up to six scheme participants per session). Within both settings, scheme participants were asked to contact the researcher if interested in participating. All scheme participants expressing an interest in participating in the study took part.

Scheme referrers are represented by GPs and practice nurses recruited using the following mixed sampling approach. An email detailing the study aims and request for participants was sent to all GP leads within four LAs via a coordinator. Later, individual practices within wider LAs were also contacted using existing networks to boost recruitment activities. Scheme referrers interested in taking part in the study were asked to contact the research team after which study information sheets were distributed.

### Ethics, consent and permissions

 Ethical approval for this study was obtained from Cardiff University’s School of Social Sciences Research Ethics committee (SREC/2163) and Cardiff University School of Medicine (SMREC number 19/43). Study information sheets were distributed to participants in the weeks before interviews took place. All study participants provided written informed consent for participating, including audio recording of interviews and use of anonymised quotations. All participants were assured that participation was voluntary and that study withdrawal was possible up until publication.

### Data collection

 Each participant was offered the opportunity for a face-to-face interview or alternatively a telephone interview for their convenience. Author Morgan conducted all scheme deliverer and referrer interviews and co-author Lewis conducted all patient interviews. The length of interviews varied according to the stakeholder subgroup; referrers (range 10-22 min), deliverers (range 24-98 min) and participants (range 6-42 min). At the beginning of each interview, the interviewer provided a brief recap of the study purpose, outlined the format of the interview and reiterated assurances of confidentiality. For each group of stakeholders, a semi-structured topic guide comprising open-ended questions and prompts was used (Additional file 1 Table A1). The following broad topics were covered for referrers: frequency of and reasons for referral, facilitators and barriers to referral, patient journey through the scheme and process for referral. Scheme deliverer guides covered: referral rates, types of referrers and engagement of surgeries. Topics covered within patient interviews included: experiences of the referral process and reasons for uptake. Data collection took place between May 2017 and October 2019.

### Data analysis

All interviews were digitally recorded, anonymised and transcribed verbatim. NVivo 12 software was used to assist with data management, indexing, and coding. Themes were derived following the iterative phases of our thematic analysis approach [19].

Several opportunities for a sense-check of the data were undertaken in order for participant groups and stakeholder representatives to feedback on the initial data findings. Summaries and initial themes were presented at a series of national stakeholder groups attended by scheme deliverers and to patient representatives from the Involving People Community (*N *= 2) who had participated in the scheme previously but had not taken part in the study. At each event, participants were asked for their feedback and encouraged to make further comments.

All analyses and double coding were undertaken by two co-authors (Morgan and Lewis). To begin, one third of transcripts for each dataset were independently read and coded before both researchers met to discuss their understanding of the data and agree initial themes. The key points of coded data were indexed and summarised thematically. Data saturation was achieved by the coders with five key themes identified. Each theme is represented by verbatim quotes to represent a range of views.

In order to map the interplay of results across multiple levels of the social system, theme data were grouped into layers of the Socio-ecological Model [20]. Data are displayed from the individual, interpersonal and community, organisational and societal level, with key facilitators and barriers to scheme referral presented.

## Results

### Characteristics of participants

A total of 50 individual stakeholders took part across 22 LAs in Wales. These included 9 scheme referrers (across 7 general practices and 3 LAs), 22 scheme deliverers (across 21 LAs) and 19 scheme participants (across 4 LAs). Table 1 displays an overview of recruitment numbers and an additional file provides a breakdown of participant characteristics (see Additional file 2, Tables A2.1, A2.2 and A2.3). On average scheme deliverers had 7 years of experience in the role (range 1-11) and 26.32 % (*N *= 5) patients had requested a referral to the scheme.

### Key themes

Five key themes were developed from analyses; referrer characteristics, geographical disparities in referral and scheme access, reinforcements for awareness of the scheme, patient characteristics and processes and context underpinning a referral.

**Fig. 1:**
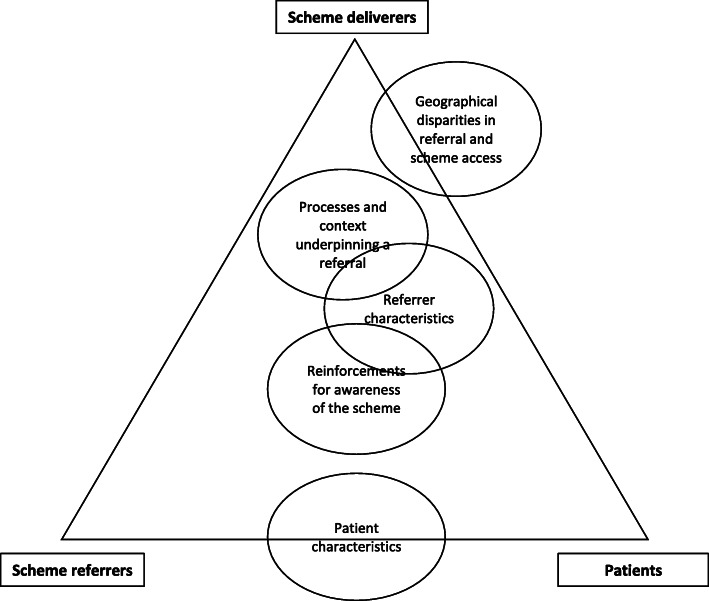
Overview of emerging themes from all stakeholder interviews

The relationship between themes and stakeholders is depicted in Fig. 1, with themes common to both (i.e. along the same adjoining line) or all (i.e. in the middle of the triangle) shown. For example, the ‘Geographical disparities in referral and scheme access’ theme was identified from both scheme deliverer and patient data. The positioning also signifies how strongly that theme was discussed (e.g. ‘patient characteristics’ is located at the mid-point between scheme referrers and patients as both groups discussed this equally, while ‘Geographical disparities in referral and scheme access’ was mainly discussed by deliverers) as agreed by the two data coders.

Results from patient data were similar across the four LAs therefore no differentiation is highlighted in the presentation of themes.

**Table 1:** Stakeholder recruitment for 1:1 interviews

Stakeholder	Local authority (N)	Recruitment (N)		Format (N)	
		Participated	No response	Face-to-face	Telephone
**Scheme deliverers**	21	22^a^	1	5	17
**Patients**	4	19		16	3
**Scheme referrers**					
General Practitioner	3	5		1	4
Practice Nurse	1	4		0	4

^a^2 part-time coordinators interviewed in one LA

**Fig. 2:**
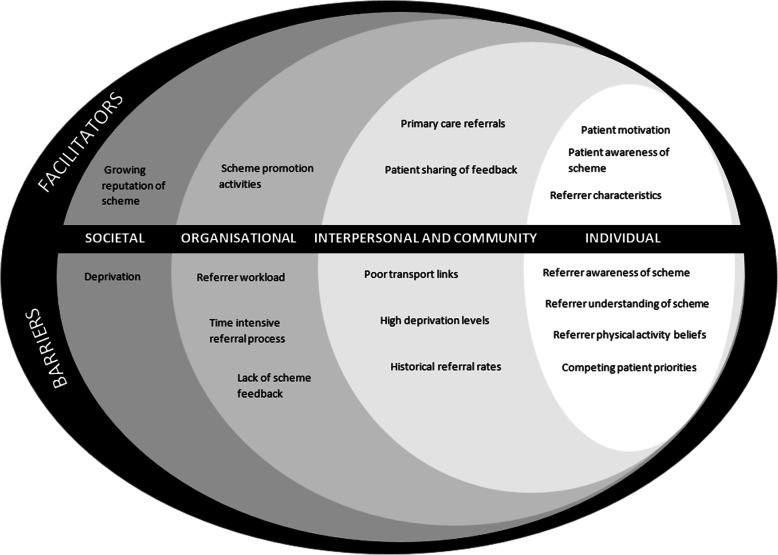
Factors influencing referral rates to NERS across the Socio-ecological Model

Figure 2 displays a summary of factors influencing scheme referral across the Socio-ecological Model. As shown, facilitators and barriers to referral were identified across all levels. Scheme referral was dominated by theme data at the individual level, reflecting underpinning characteristics of both the referrer and patient (Themes 1 and 2). Data specific to the scheme are displayed under the organisational level.

### Theme 1: Referrer characteristics

An overarching theme identified across all stakeholder datasets was the referrer’s characteristics (Table 2), with a number of subthemes determining whether a referrer was likely to engage with the programme referral routes. This was perceived to be influenced by the referrer’s demographics, lifestyle and their perceptions and awareness of the scheme.

**Table 2:** Referrer characteristics – sub-themes (Theme 1)

Subtheme	Deliverer	User	Referrer
Interpersonal traits	The age of the doctor makes a big difference, the older the GP generally, they’re not interested, or they don’t refer as many, it’s more the young, progressive doctors that do it, and so, and they understand the importance of it anyway. (21)		
	I think it definitely goes on what especially the GP’s are into themselves. This is what I found anyway, you know the GP’s that are into sport themselves, because they’re the ones that are referring through you know. (22) It’s down to the practice nursing staff, if they have very high BMI’s, I found that a lot of them won’t refer because it’s things like, well if I’m overweight, then you are overweight, but you’re telling me to go, why don’t you go? (21)		
Perception of scheme	It’s usually if it’s just that GP’s belief, you hear the odd time, patients come into the Leisure Centre and they’ve gone, “oh we didn’t know about it and the GP wasn’t keen about sending us, but it was only because we pushed about”, so it’s usually the barriers being the odd GP, who’s not been keen. (1)		I think you’ve got be able to see the value in it, you know. So you’re not going to do something you don’t think is, is worthwhile. And I think this is worthwhile. (1)
Awareness of scheme	But also the fact that the scheme is better known now, means that more health professionals are likely to refer into it… (7) All the GP’s should be aware of us. We do have them occasionally saying they’ve never heard but they tend to be new GP’s coming into post or locums. So that tends to be the issue. (12)	No, I know my doctor, when I told him I was waiting, he didn’t seem to know anything about this referral card, and he’s the head of the practice, he’s been there, you know… for a long time. (9)	I haven’t had interaction with it at all. So when I started at my previous surgery I assumed that there was an exercise referral so I had asked my colleague, and she said, “Oh we don’t do that anymore.” So I just took her word because I was brand new to practice nursing and obviously didn’t know anything. I was only relying on my colleague to let me know what was happening. So I took it as … ‘oh it can’t be going anymore. It must have ceased’, just finding this quite frustrating because there were a lot of patients that would’ve benefited from being referred. (5) One thing I find hard is not knowing what they will do … just as comparison, like the expert patient program for diabetes with the dieticians. They explain to you exactly what goes on in each session and what the patient can expect, and how long it lasts, whereas with this one I don’t feel I know enough about it. (7)

Scheme deliverers perceived referrer characteristics as a key determinant which influenced referral rates. These centred around interpersonal traits whereby younger referrers were considered more engaged and aware of the benefits of physical activity, whereas older referrers were perceived to be more likely to focus on traditional treatment options such as prescribing medications. The alignment of referrer characteristics with the scheme was also discussed as both a facilitator and barrier to referral. Referrers who were known to have an active lifestyle were perceived to be more likely to refer to the scheme. Referrers with a higher BMI were considered less likely to refer due to a perceived fear of judgement. Recognising the value of the scheme as a worthwhile experience for patients was also discussed as a determinant of referral.

The majority of deliverers highlighted the growing reputation of the scheme since the original trial and did not perceive a lack of awareness among referrers as a barrier to referral. Albeit in some instances, newer staff members or locum/freelance general practitioners were identified as having a lack of awareness. Patients however, described experiences whereby there was a lack of, or in some cases an absence, of scheme awareness among their health professionals, including among more established members of staff. Referrer accounts highlighted the complexities when considering scheme awareness, with consideration given to the both awareness of the scheme’s existence and awareness of the scheme’s nature. For instance, while some referrers acknowledged their overarching awareness of the scheme, they also stressed their lack of understanding of what the scheme actually entails, with the latter being identified as a key barrier to referral. One health professional emphasised the disparity of awareness amongst GP practices, describing their frustration in not knowing whether referral to the scheme was offered at all at their new practice having recently moved surgeries.

### Theme 2: Geographical disparities in referral and scheme access

Location, of the referrer and scheme delivery, was identified as a barrier to referral, with key factors concerning area deprivation and scheme accessibility, while some interviewees described variability in referral rates that they felt unable to explain (Table 3). This theme was largely dominated by data from scheme deliverers, with some input from patients on scheme accessibility.

**Table 3:** Geographical disparities in referral and scheme access – sub-themes (Theme 2)

Subtheme	Deliverer	User	
Deprivation	There are small pockets, where it’s been a constant challenge to do so, and for me it’s the south of the Authority … and yet this is an area that is identified as inequalities and greatest demand, and yet it’s so tough to try and get the health professionals to engage better and we’ve got some of the better services available, but they’re not utilised you know, in a way that they could be a bit more positive. (8)		
Accessibility	There are some practices that are thirty miles from their local leisure centre. So, I think that plays a big part in referrals rates as well really. I think our referrals to our main leisure centres within the towns are very good and probably too many referrals, and then from a rural area they are few and far between. (11) There’s quite a lot of rural areas around all that as well so, public transport isn’t very good … … It’s not easy for some of the people, if they don’t drive they either rely on friends or family or try and make the public transport work, or they don’t come. (17)		Yes, it could be [accessible by public transport]. What it is and what bus I’ve got to catch, I haven’t got a clue… Well, if I had to use public transport, I’d most probably nip over and use (town) facilities instead, because that’s nearer to me, but I prefer the atmosphere… (13) From where I’m living now, I’d have to catch a bus into town and a bus from town down to where the centre is … but it’s not very convenient. In fact, they’ve just taken a bus off from us in (suburb) that went down to the hospital, and from the hospital into town. They’ve taken that off. (18)
Accepted variation	GPs, there’s some very active, and you know, are very active referrers, as GPs. Some GPs are not, so that’s just the way it’s always been. And in some areas you know, there’s no referrals, there are absolutely none, but in some areas it’s very high, which then creates an imbalance. (5)		

Engaging GP practices within areas of deprivation in referring patients was recognised as a key challenge for scheme deliverers. For some, this was an inherent challenge as deliverers recalled a persistent a lack of referrer engagement despite an increasing number of services available within pockets of deprivation.

Poor transport options and greater travel distances to scheme facilities were perceived by both the patient and scheme deliverer as barriers to referral. While leisure facilities within town centres often received a high number of referrals, deliverers argued that even when a referral has been made, patients often will not attend the scheme if public transport is not a viable option. Requiring multiple bus journeys, uncertainty of travel timetables and disruption to routes were cited as barriers. A high proportion of patients reported using their own mode of transport to attend sessions, with some travelling further afield to access a scheme with more desirable facilities or class timetables.

In some LAs, scheme deliverers described an imbalance between referral rates across different areas within a LA. This imbalance was perceived a persistent challenge and highlighted inequity of scheme provision within a LA. Scheme deliverers were unable however, to pinpoint any underpinning reasons for this, simply reiterating that some GPs were frequent referrers while others had never made a referral to the scheme.

### Theme 3: Reinforcements for awareness of the scheme

All stakeholders described how awareness of the scheme among referrers and patients impacted upon referral rates (Table 4). Subthemes identified efforts to raise awareness among GP surgeries, increased patient awareness leading to self-referrals and wider referrals from primary care. The experiences described by stakeholders within this theme were often intertwined with accounts of the broad eligibility criteria for a scheme referral.

**Table 4:** Reinforcements for awareness of the scheme – sub-themes (Theme 3)

Subtheme	**Deliverer**	**User**	**Referrer**
Instructor efforts	We do go out now and again to GP’s. Try and target GP’s who are not referring or maybe we’re getting inconsistent referrals from them. Uncompleted forms, that kind of thing. So it tends to be more of a fine tuning exercise now rather than actively recruiting. We’ve already done that. (12) It’s been continuous having to knock on doors of medical professionals, keep it in the forefront of their minds and try and keep them referring. So we’ve done that by quality of service but what we really need is help from the top coming down. (20)	Well, the fact that they are very good at what they do (3)	
Self-referral	So I know there is a large number of our referrals actually make a point of going to their GP or Practice Nurse and requesting that they’re referred. (18) The other element of it is that as it’s more well-known, and people know that it is a cost effective way of exercising, so it’s cheaper than your traditional gym membership, I think that has a big impact on how many people want to be referred as well. (7)	I think I asked the nurse because I saw a sign on the wall outside the surgery that there was some kind of, exercise referral group. And by then I was willing to try anything to try and help resolve my problems. (19) …certainly, from the managing your own conditions there is a bit of a scheme in the borough for managing long term health conditions and people used to be able to self-refer to but I think it’s a consultant referral now. (2)	So I would refer anybody who asked for it. I don’t think I would ever refuse someone who has requested it… think you can almost always find a reason to … you know if someone asks for it, it’s very difficult not to find something to send them in for so … I mean if they were 30, completely fit and well, no anxiety and depression and just wanted to go to the gym for free, perhaps I would suggest there might be other routes to go down but I haven’t had that scenario. (1) I don’t know whether patients can self-refer. I don’t think that they can but I think if they could self-refer that would be more beneficial for them, so that they didn’t have to come see us and, and to get a referral from, from primary care. (4) I probably don’t push it enough even though I think it’s a really good idea. Definitely I think exercise is the answer to most of the problems we see, a lot of them anyway. I probably don’t push it enough. I probably wait for people to ask for it. … … it’s rare that I look back in notes and that I see doctors have, say, encouraged to go on exercise program scheme. It’s usually a patient wants to go on, so I’ll refer. (2)
Primary care feeder	I think it’s just because it’s a natural progression from people who have gone to see the physio for exercise, and it’s a natural progression, a natural exit route for them to do really, so I think it makes sense, whether they’re the right people to be referred or not, I don’t know? But we do have a high percentage of them coming through. (22) I think there’s definitely a case sometimes of let’s pass that on to somebody else. I think physiotherapy departments are under massive pressure. I get a feeling physiotherapy in (area) wouldn’t even be able to cope without us. (12)		

The majority of scheme deliverers described their efforts to engage with referrers, with some areas investing more time and effort than others. Activities centred on raising awareness of the scheme, providing familiarity and building trust with health professionals. While deliverers highlighted the endorsement of such efforts by the overall scheme lead, they also emphasised the time investment required and persistent balancing act for scheme promotion and scheme delivery. One coordinator suggested that the onus for enhancing referrer awareness should be placed on national bodies as opposed to a responsibility for local teams.

All stakeholders identified patient awareness of the scheme as a facilitator to referral, with an increasing number of patients requesting to be referred. In some instances, this was perceived to be linked to the subsidised cost of the scheme with patients seeking an opportunity to access leisure facilities cheaper than traditional memberships. That said, scheme referrers acknowledged that almost any patient could be referred to the scheme given the broad eligibility criteria yet one referrer explicitly stated they would refrain from referring healthy patients seeking a discounted membership. Patients described their request for a self-referral as a way to manage their own health conditions. While some deliverers highlighted instances whereby individuals have asked to join the scheme at the leisure centre (and were subsequently signposted to their GP for a referral), one referrer queried whether patients could self-refer directly to the scheme and bypass sign-off from a health professional. This was identified as a desirable time saving approach for referrals.

The expansion of the scheme to encompass condition-specific pathways has led to greater awareness among health professionals outside of GP practices and was seen as a reason for increasing referral numbers. Scheme deliverers discussed how primary care health professionals view the scheme as a natural progression for patients, with follow-on referrals from physiotherapists for example. It was also stated however, that in some instances primary care health professionals utilise the scheme to ease pressure on other local over-subscribed services.

### Theme 4: Patient characteristics

Patients and scheme referrers identified characteristics relating to the patient at the time of referral as key to scheme referral, these included the patient’s motivation and priority setting (Table 5).

**Table 5:** Patient characteristics– sub-themes (Theme 4)

Subtheme	User	Referrer
Motivation	I suppose my motivation, really, to do something, that I was actually asking for that, and my reasons for it. But I remember saying, because I thought it was more appropriate for me so I sort of self-selected it really. (2)	Well, I suppose, [pause]. You know. Actually getting the patient to physically sign is probably a good thing isn’t it, you know, people who are motivated … are you more likely to get the motivated people, who actually you know do go for it, whereas those that are not so well motivated they, … they’ll say, “No, I don’t want to sign the form.” (9)
Priority setting	I suppose for something like this which may be seen as a “it would be helpful but it’s not life-saving”, you know, so it’s not a huge priority. (6)	I think a lot of patients as well don’t see the value in it, unfortunately. Its way down on their priority list. You know a lot of patients we see, cos it’s quite a deprived area, if you say ‘exercise’ to them it’s like, you know. They almost find it laughable I think because they think they’ve got so many other bigger problems than exercises. They can’t really see how it would help them. (2)

The motivational status of a patient at the time of referral was discussed as both a barrier and facilitator to referral. High-levels of motivation were often accompanied by a self-referral (i.e. asking the referrer for a referral) whereas referrers had also encountered situations whereby patients with lower levels of motivation had refused to approve the referral despite referrer encouragement. Motivation was also acknowledged as a referral facilitator by a patient who described their own experience of ‘self-selecting’ themselves into the scheme by asking for a referral.

Some patients perceived the scheme as a desirable option as opposed to a necessity for improving their current health, with one highlighting that the scheme ‘would be helpful but it’s not life-saving’. This notion was similarly acknowledged by referrers who highlighted that for some patients, particularly those from deprived backgrounds, awareness of the scheme and recognition of its value are likely to be lacking given the greater number of challenges facing such communities. One referrer emphasised this point by describing ‘exercise’ as almost laughable to patients who perceive many other problems to be more prominent in their lives.

### Theme 5: Processes and context underpinning a referral

The extent to which context and processes constrain or facilitate a referral were highlighted by all stakeholders, with key processes, referrer workloads and increasing referral pathway options discussed (Table 6).

**Table 6:** Processes and context underpinning a referral – sub-themes (Theme 5)

Subtheme	Deliverer	User	Referrer
Referral procedures	I think it fluctuated because originally we had booklets, and the referral form was paper. We’d leave paper pads in the practices and then say the practice nurse would keep the pad with her. So whenever she was running clinics like the pre-diabetic clinic or asthma clinic, or whatever, she’d then quickly fill the paperwork in and they’d be posted through to us or we’d go and pick them up. So sometimes then they couldn’t find a pad, the pad would get mislaid, you know, they’re all very busy themselves with their jobs. That then was replaced, we’ve now got this electronic referral system so they can email referral forms through to us. Again that has had some problems because if the practices haven’t got access to a computer there and then if they’ve got any clinics, they don’t necessarily get the referral forms through to us. (7)		You’d hope that with what we’ve got technologically now you could potentially, bring up the form that self populates in your computer system and email it off to a central address. That would be … the time it takes… to do stuff. It’s incredibly important because there’s so much time pressure all the time dealing with people that it is something that you can do relatively quickly. You don’t want to be spending time entering patient details. You’re already in the patient’s file. You don’t want to be putting details of what medications they’re on. They’re already on the system. You basically want to be saying, “This is the reason I’m sending the person to you. This is what I want help with.” … The rest of it you want to be done. And then you don’t want to be printing it off and taking to … or sending it, you know? You want it to be send-able from the screen you’re on and sent away and you know it’s got there. That’s what you want and if it works that way more referrals will happen for sure. (6)
Feedback mechanisms	Once they get up to speed and once we’ve had feedback from the people that have actually participated and hopefully most of it’s been pretty positive, they’ve bought in, they understand and they feel it’s a safe environment as well to send their patients to. (21)	I don’t know, but if they collect this information about how many clients they got on, and the feedback from the instructors, I know is just registered on the computer and whether it’s used or not I don’t know. But if they did disseminate that to the doctors and showed, cos I’m sure it shows in very positive light, how the scheme works, if that was disseminated to the doctors on a regular basis of the clients from the various practices who’ve gone there, I don’t know if any feedback exists. … the only feedback my doctor, my GP had, was feedback that I give him, and I give, I always give him what I felt was really high positive feedback. And he was quite complimentary on that, as well. (19)	Perhaps one of the biggest issues with it is I feel like I don’t know really know in detail what happens to people when they are there and, for whatever reason, because I’ve not had many people coming back telling me about it, that seems a bit … and not quite knowing what you’re putting people forward for is a barrier I think. … Feedback about how people do would help encourage you to do it. So then you can confidently say to the person, “This is what’s going to happen and I know this works because I’ve had several people it’s worked well for,” that sort of stuff. (6)
Workload	We’ve got a very good GP who sits on our steering group and he was very honest and he said, you know, you aren’t our priority when a patient comes through. Our priority is to diagnose and prescribe and then sometimes yours is seen as a nice to have service at the end. (7)		And if there’s less time in the appointment and you just haven’t got that time to talk at length about, wellbeing, you might just have spent an awful long time talking about say diabetes, might have spent a long time talking about the medication, getting the medication changed, talking to the GP. By the time you’ve done all that 15 min has gone and you haven’t got time then to talk about it, you know? (5)
Expanding pathways	As the referral scheme has moved on and we’ve introduced level four conditions, for example, falls, stroke, weight management, cancer, mental health, pulmonary, cardiac, what’s happened is referrers have referred to the correct pathway. So for example we may have had somebody referred on generic who, when you get them in for a consultation, needs level four intervention and not generic. So what has happened, the increase as we’ve added a new level four it’s opened the doors for health professionals to refer to the correct pathway so, yes, level threes have increased and level fours have increased. (3)		

Scheme deliverers noted that over the years there has been an introduction of changes to referral form completions. They described how some areas are continuing to complete paper-based referral forms while others have trialled an electronic system. This process was highlighted as a key barrier by a number of deliverers and referrers with accounts of data entry errors, duplication of information and an overall time intensive activity for referrers. Many referrers expressed the need for an automated system which would reduce both the time and effort required when making a referral.

Besides the time required to physically complete a referral form, referrers noted the constraints of typical GP practice appointments as a key challenge to referral. Most scheme referrers stressed the difficulty of meeting competing demands within an appointment, often having to balance wider health complaints and the needs of a patient in a short space of time. This was also recognised by scheme deliverers who acknowledged that referrers are often unable to prioritise a referral. One scheme deliverer recalled wider referrer feedback which highlighted that the role of the health professional is to diagnose and prescribe, with NERS seen as a ‘nice to have service’ rather than standard prescription method.

A key referral facilitator described by patients and referrers, was instances whereby a patient shared their positive scheme experiences with their referrer. This was highlighted as a key motivator for making future referrals. That said, many referrers described the absence of formal scheme feedback as a key barrier to referral. Coupled with the uncertainty of not fully understanding what patients do at the scheme, a lack of feedback meant that referrers were unsure as to whether referred patients attended and if they did, when and how the scheme had impacted upon their health since. As such, the introduction of a formal feedback mechanism was requested as a facilitator for future referrals.

Deliverers acknowledged the expanding programme, with a number of new specific health referral pathways introduced over the years. As such, it was noted that referrers had begun to select the appropriate referral pathway whereas before the referral would have been via the generic pathway. Subsequently, while the overall demand for the scheme continues to increase, any decrease observed in generic referral numbers was perceived as a consequence of expanding pathways and allocations to the most appropriate referral pathway.

## Discussion

This qualitative study explored experiences of referral within NERS, an ERS which has been embedded in routine practice for over ten years. Reflecting perspectives of the referrer, scheme deliverer and patient, our findings highlight several barriers and facilitators to scheme referral, with factors spanning across the Socio-ecological Model. Major themes identified included: referrer characteristics, geographical disparities in referral and scheme access, reinforcements for awareness of the scheme, patient characteristics and processes and context underpinning a referral.

Consistent with earlier research among NERS professionals [16] and wider literature [21, 22], barriers to referral included physical activity beliefs of the referrer alongside certain referrer demographics and characteristics. Typically dominated by perceptions of scheme deliverers, various referrer characteristics were described as key determinants to referral decisions. Unlike findings within the earlier RCT [16], the age of a referrer was identified as an influential referral factor in the current study, with accounts of higher referral rates among younger GPs and a traditional stance of prescribing medications among older health professionals. Notably, wider research has found higher rates of referrals among an Australian-based exercise scheme [23] and referrals for counselling behaviours [24] among younger physicians. Valete and colleagues [25] also reported a greater appreciation for the values of aerobic exercise among younger physicians. Such findings could reflect developments of recent training programmes as Chatterjee et al., [26] noted greater knowledge of physical activity guidelines and greater confidence in using physical activity tools among younger GPs.

An overarching barrier to referral was a lack of awareness, both awareness for referral capability and awareness of what the scheme entails. Patients recollected experiences whereby referral requests were met with referrer uncertainty while one health professional expressed her frustration as to not knowing if referral was an option across all GP practices. All health professionals strongly emphasised their lack of understanding of what happens to a patient following a referral, with an inability to describe the subsequent patient contact process, the length of the scheme or what exercises the scheme entailed. Leemrijse and colleagues [27] found similar findings among Dutch exercise referrers while limited knowledge and a subsequent lack of confidence among practitioners have previously been shown to impair the ability both to discuss and prescribe physical activity [28, 29]. Of note however, scheme deliverers did not consider awareness for referral capability as a barrier, on the contrary many described their activities dedicated to keeping referrals at the forefront of health professionals’ minds. For some, such efforts were considered time-intensive and there was a call for wider support to ensure physical activity promotion was firmly on the agenda for health promotion among all GPs.

In agreement with earlier accounts of NERS health professionals [16], the lack of time to introduce and discuss the scheme with patients continues to be a barrier to referral and our findings demonstrate how this barrier is exacerbated within areas of deprivation. Referrers discussed the complexity of patient health needs residing in areas of high deprivation and the difficulties of addressing multiple patient needs within a single appointment. They also acknowledged that referral to an exercise programme was likely to be considered low down on a list of priorities for patients experiencing complex needs. These findings go some way to explaining our recent reports of a widening of inequality in NERS referral and uptake rates [12] and the current frustrations expressed by scheme deliverers towards the lack of referrals from areas in greatest need. Tudor Hart stated that time is the real currency of general practice [30], yet the pressures of time are acutely felt in areas of deprivation with increasing GP stress levels [31] and documented inequalities in primary care accessibility [32, 33]. In recognition of such challenges and to address the inverse care law, varied attempts to support GPs within deprived areas are underway in the UK. For example, within deprived areas of South Wales the Academic Fellows scheme [34] adopts a bottom-up approach to support GPs to make service improvements to their local population while the Scottish Deep End project aims to lobby for change in policy and investment among the 100 most deprived populations in Scotland [35]. NHS England have recently introduced link workers, such as Social Prescribers or Well-being coordinators within GP surgeries as a means to free up GP appointment times to focus on complex conditions while giving patients time to talk through their needs with signposting to local services such as exercises classes [36]. A key intervention-specific barrier directly impacting a referrer’s time availability concerned accounts of a time intensive and disjointed referral system. It became apparent that across general practices the referral system varied depending on technology and internet access, with some areas using paper referrals and others an electronic system. Frustrations were encountered by referrers when required to manually input information from the practice database, whereas scheme deliverers acknowledged that paper referrals often did not reach them. Study findings are consistent with wider literature detailing a breakdown in communications at the referrer-scheme interface due to a reliance on antiquated communication technologies [37]. A further intervention-specific barrier was the perceived lack of scheme feedback. This finding also echoes reports of health professionals involved in the earlier RCT [16] suggesting little change to key processes since NERS has become embedded within routine practice. A similar finding has been reported previously among health professionals in England, with an expressed desire to see feedback on patient performance and commitment to behaviour change, yet the provision of feedback was not monitored as part of this ERS [17].

### Implications for practitioners, policymakers and beyond

The sustainability of health-related schemes such as ERS is important for funders and implementers alike yet there is a prevailing emphasis on implementation and effectiveness research, with the prior typically focusing on initial implementation [38]. Considering the life cycle of an ERS, it is important to understand how schemes are maintained and evolve beyond their original implementation, with a view to inform sustainability in the future [39]. While NERS continues to be sustained within routine practice on a national scale, the current study highlights that a number of referral barriers identified over a decade ago continue to persist. These barriers largely pertain to two themes; referrer characteristics and processes and context underpinning a referral. In order to address these and enhance scheme sustainability, a number of recommendations are highlighted below.

There is a clear need to introduce a sustainable approach for enhancing the awareness of both trainee and established health professionals on their ability to refer patients to NERS and of what the scheme entails. Firstly, embedding training schemes to deliver physical activity advice-giving as part of standard general practice training and continuing professional development could prove advantageous for enhancing overall scheme awareness among health practitioners [16]. For example, a national programme Moving Healthcare Professionals has been established in England to support healthcare professionals to increase knowledge and skills around physical activity and identify ways to incorporate physical activity within routine care [40]. To support sustainability, the programme adopts a whole-system approach to embed training throughout undergraduate education, postgraduate education and continuing professional development pathways [41]. Secondly, there is a need to develop accessible guidance for use and dissemination across all general practices in Wales. Guidance needs to provide a clear overview of the content of the NERS programme and what patients can expect once a referral has been made. Ensuring all GP practices have the option to provide informative materials to patients at the point of referral would enhance scheme understanding for the patient, limit the need for referrers to explain scheme features and ultimately maximise the limited consultation time. Furthermore, the availability of resources within general practices would reduce the onus on scheme deliverers to provide constant reminders and prompts. At present, information on the NERS programme is available via the provider’s website, with an overview of the programme, breakdown of the different referral pathways and a selection of patient case studies [42].

In the present study, a frequently mentioned facilitator for making a referral was a high level of patient motivation. This was often portrayed by a patient requesting a referral to the scheme with one health professional recognising that they only made referrals when requested by patients. Such findings are consistent with earlier NERS research [15, 16] and patients already motivated towards physical activity have been shown as more likely to adhere to an ERS [15, 43]. To help further address the lack of referrer time and capacity, NERS could introduce an option for patients to complete a self-referral form, an approach currently offered by other ERS in the UK [44]. This would eliminate the need for a GP appointment and in turn reduce both referrer workload and wait time experienced by the patient. That said, our findings do, however, reinforce concerns about scheme referral being limited to the worried-well [16] despite known benefits among those patients who enter the scheme for more externally motivated reasons (e.g. health professional advice) [45]. Future recommendations to increase scheme awareness among health professionals and ensure the referral process is timely, equitable and efficient are all of equal importance. Current work within Wales [46] and elsewhere in the UK [47] recognizes the role of exercise within the landscape of social prescribing, particularly as a strategy for tackling health inequities while reducing the pressures on the National Health Service. In this instance, individuals could access services in the voluntary and community sector, rather than relying on health professionals to provide a solution.

To help overcome intervention-specific barriers discussed above, there is a need to improve both the efficiency of communication channels and information management across the referral system. One avenue is to combine the use of an electronic referral system with electronic medical records collated at each general practice [37]. Such an integrated system could enable self-populating fields at the point of referral and include feedback mechanisms which would advance information sharing and improve the efficiency of referral submissions. Furthermore, with the integration of feedback mechanisms, referrers could receive automated updates on a patient’s progress across the scheme. In turn this would reduce referrer uncertainty surrounding patient progress, provide an additional layer of reinforcement for patient engagement and help to improve referrer confidence for future referrals.

### Strengths and limitations

This qualitative study was a component of a larger NERS evaluation which aims to provide insights into an established, national ERS ten years on since findings of a RCT. A key strength of this paper is the triangulation of data from three stakeholder groups, providing views across the referral process. Topic guides were influenced by earlier NERS research [15] and scheme deliverer data represented all but one LA across the country. Unlike earlier research based on the trial phases of NERS, the current research is founded on an established service and as such views are not influenced by initial enthusiasm for a new service or the need to randomise patients to the scheme.

There are however a few limitations. Given the convenience sampling method used to recruit both health professionals and patients, it is possible that views represent a biased population. The difficulties encountered while recruiting health professionals further emphasises the complexity of engaging referrers with limited time capacity yet, the geographical spread of health professionals represents areas of low and high deprivation. While scheme deliverers identified referrer beliefs as a determinant for referral, we did not ask health professionals about their personal views of physical activity, a factor which may play an important role in the decision of whether to refer or not. In addition, we were unable to capture the viewpoints from patients who have been referred to NERS but have since not taken up the scheme.

## Conclusions

NERS is an established, evidence-based programme which continues to support thousands of patients across Wales each year. The results of this study demonstrate the breadth of barriers across the referral process and go some way to explaining recent reports of a widening of inequality among referrals. This study provides evidence that could inform the further development of NERS to ensure the referral process is timely, efficient and equitable. These findings have since informed the design and development of a future NERS national database.

## Supplementary Information

**Additional file 1.**

**Additional file 2.**

## Data Availability

The interview questions are outlined in Additional file 1 Table A1. The interview transcripts generated during the current study are not publicly available due to ethics requirements.
