# Supplementary material for: From a research trial to routine practice: stakeholders’ perceptions and experiences of referrals to the National Exercise Referral Scheme (NERS) in Wales
Source: BMC Health Serv Res. 2021 Nov 13;21:1232. doi: 10.1186/s12913-021-07266-7 (PMC8590360; doi:10.1186/s12913-021-07266-7)
Supplement: Supplementary file 1 — Additional file 1. [file 12913_2021_7266_MOESM1_ESM.docx]

**Additional file 1 Interview Guides**

Table A1 Interview Guides

| **Scheme referrers**  Can you talk me through any interactions you have had, if any, with the NERS team in your area?  How often do you refer patients to NERS, if at all? How do you determine which patients to refer (or not refer) onto the NERS scheme? (instances of self-referral)  How do you introduce the scheme to patients?  Do you yourself complete the referral form? (paper, electronic, staff nurse)  What happens once you have referred the patient to the NERS team? (any mechanism for feedback or communication)  Do you get instances of re-referral?  What are the main reasons for your referral onto the scheme?  From a practitioner point of view, what are the facilitators and barriers when referring to a health scheme like NERS? |
| --- |
| **Scheme deliverers**  During your time in this role, what have referral rates been like? Have there been any fluctuations in rates? Any reasons behind this?  Who do you get most of your referrals from? (i.e. GPs, practice nurses, physios etc…).  Are any practices more engaged and more likely to refer than others? Explore why that might be. |
| **Scheme Users**  How was the exercise scheme introduced to you? Had you already heard of the exercise scheme before this?  What factors helped you to make your decision to accept being referred to the scheme?  Why do you think GP surgeries are choosing to refer patients into an exercise scheme such as this one? What do you think motivates / discourages them to refer patients?  Are there any factors that you can think of that affect a person’s likelihood of being referred into the scheme? |
