# Supplementary material for: From a research trial to routine practice: stakeholders’ perceptions and experiences of referrals to the National Exercise Referral Scheme (NERS) in Wales
Source: BMC Health Serv Res. 2021 Nov 13;21:1232. doi: 10.1186/s12913-021-07266-7 (PMC8590360; doi:10.1186/s12913-021-07266-7)
Supplement: Supplementary file 2 — Additional file 2. [file 12913_2021_7266_MOESM2_ESM.zip › Table A2.1.docx]

Table A2.1 Scheme referrer characteristics

|  | **ID** | **Gender** | **Role** | **LA** | **LA**  **deprivation*** | **General Practice (ID)** |
| --- | --- | --- | --- | --- | --- | --- |
| **Referrer** | 1 | M | GP | 01 | low | 1 |
|  | 2 | M | GP | 01 |  |  |
|  | 4 | M | GP | 02 | high | 3 |
|  | 9 | M | GP | 02 |  |  |
|  | 3 | F | PN | 03 | low | 2 |
|  | 5 | F | PN | 03 |  | 4 |
|  | 6 | M | GP | 03 |  | 5 |
|  | 7 | F | PN | 03 |  | 6 |
|  | 8 | F | PN | 03 |  | 7 |

*based on the percentage of Lower Super Output Areas within each local authority which are ranked in the most deprived 50% of LSOAs in Wales (low is below the national average (50%) and high above). M= male, F= female, GP= General Practitioner, PN= Practice Nurse, LA= local authority
