# Supplementary material for: From a research trial to routine practice: stakeholders’ perceptions and experiences of referrals to the National Exercise Referral Scheme (NERS) in Wales
Source: BMC Health Serv Res. 2021 Nov 13;21:1232. doi: 10.1186/s12913-021-07266-7 (PMC8590360; doi:10.1186/s12913-021-07266-7)
Supplement: Supplementary file 2 — Additional file 2. [file 12913_2021_7266_MOESM2_ESM.zip › Table A2.2.docx]

Table A2.2 Scheme deliverer characteristics

|  | **ID** | **Gender** | **LA deprivation*** |
| --- | --- | --- | --- |
| **Deliverer** | 1 | F | Low |
|  | 2 | F | Low |
|  | 3 | F | Low |
|  | 4 | M | Low |
|  | 5 | M | High |
|  | 6 | M | High |
|  | 7 | F | High |
|  | 8 | F | Low |
|  | 9 | M | Low |
|  | 10 | M | High |
|  | 11 | M | High |
|  | 12 | M | High |
|  | 13 | M | Low |
|  | 14 | M | High |
|  | 15 | F | Low |
|  | 16 | F | High |
|  | 17 | F | Low |
|  | 18 | M | Low |
|  | 19 | M | Low |
|  | 21 | M | Low |
|  | 22 | M | High |
|  | 23 | F | Low |

*based on the percentage of Lower Super Output Areas within each local authority which are ranked in the most deprived 50% of LSOAs in Wales (low is below the national average (50%) and high above). M= male, F= female, LA= local authority
